# Honors to Dr. Henry Reed Hopkins

**Published:** 1909-05

**Authors:** 


### Honors to Dr. Henry Reed Hopkins.

THE Medical Society of the County of Erie, under the presidency of Dr. Charles A. Wall, did itself great credit in the reception tendered to Dr. Henry R. Hopkins, at the University Club on the evening of April 19, 1909. The exercises belonging to a regular meeting of the society were dispensed with and the ceremonies incident to the reception substituted.

An appropriate program had been planned which was carried out without hitch or friction. Dr. Hopkins had served the society some forty years as member and chairman of the board of censors; he had taken the initiative in the creation of a state board of medical examiners; he had been the first cause of clearing the city of Buffalo of quacks and criminal practitioners; he was the 97th president (1903) of the medical society of the State of New York. For these and many other reasons, not essential to mention now, it was considered proper to recognise Dr. Hopkins's services to the profession and the people at large in a public manner.

First, a number of addresses were delivered. Dr. Charles G. Stockton, the reigning president of the Medical Society of the State of New York, spoke of Dr. Hopkins's activities in promoting the scientific work of that body, of his labors for many years on the committee of hygiene, and humorously alluded to the attack of "nostalgia" which developed when the society and the association were amalgamated. Dr. Stockton spoke in his usual happy vein.

The next speaker was Dr. William Warren Potter, president of the State Board of Medical Examiners, who reviewed Dr. Hopkins's work in relation to the establishment of the board, which marked the beginning of higher medical education in this state. Mr. John Lord O'Brian, United States Attorney for the Western District of New York followed with a clever and humorous address concerning his relations with Dr. Hopkins, while he, Mr. O'Brian, was counsel for the Medical Society of the County of Erie.

Dr. DeLancey Rochester dealt with Dr. Hopkins's bold stand at different times in protecting the people from medical fakirs and mountebanks. Dr. P. W. Van Peyma spoke of the part the distinguished guest played in creating midwifery legislation, also in the interest of the people. Dr. Edward Clark, formerly president of the Medical Society of the County of Erie, dealt with questions of legislation in which both he and Dr. Hopkins had together taken an active part. Dr. William C. Phelps, a veteran in professional work, spoke for the profession at large.

At the close of the speechmaking, a silver loving cup, suitably engraved, was given to Dr. Hopkins. He responded in an appropriate speech thanking his colleagues for their kind words and the token of their appreciation of his work.

A collation then was served at which the good fellowship continued. And so ended a most enjoyable evening.

